# Immunotherapy for Aggressive and Metastatic Pituitary Neuroendocrine Tumors (PitNETs): State-of-the Art

**DOI:** 10.3390/cancers14174093

**Published:** 2022-08-24

**Authors:** Tiziana Feola, Francesca Carbonara, Monica Verrico, Rosa Maria Di Crescenzo, Francesca Gianno, Claudio Colonnese, Antonietta Arcella, Dario de Alcubierre, Silverio Tomao, Vincenzo Esposito, Felice Giangaspero, Giuseppe Minniti, Marie-Lise Jaffrain-Rea

**Affiliations:** 1 Neuromed IRCCS, 86077 Pozzilli, Italy; 2 Department of Experimental Medicine, La Sapienza University of Rome, 00185 Rome, Italy; 3 Department of Biotechnological and Applied Clinical Sciences, University of L’Aquila, 67100 L’Aquila, Italy; 4 Department of Radiological, Oncological and Pathological Sciences, La Sapienza University of Rome, 00185 Rome, Italy; 5 Department of Advanced Biomedical Sciences, University of Naples, Federico II, 80131 Naples, Italy; 6 Department of Neurology and Psychiatry, La Sapienza University of Rome, 00185 Rome, Italy; 7 Department of Medicine, Surgery, and Neurosciences, University of Siena, 53100 Siena, Italy

**Keywords:** aggressive pituitary tumors, pituitary carcinoma, pituitary neuroendocrine tumors (PitNETs), temozolomide, radiotherapy, PDL1, immune checkpoint inhibitors, tumor mutational burden, mismatch repair, immune-related adverse effects, case report

## Abstract

**Simple Summary:**

The treatment of aggressive and metastatic pituitary neuroendocrine tumors (PitNETs) refractory to current therapies, including temozolomide, is challenging and hopes are relying on personalized therapies. Immunotherapy based on immune checkpoint inhibitors (ICIs) is a revolutionary tool in oncology. This study was stimulated by our recent experience with a metastatic Pit1-derived PitNET expressing PDL1 and showing a remarkable response to pembrolizumab. After a detailed review and critical analysis of a total of 13 cases reported so far (12 from the literature), a significant clinical benefit of ICIs was clearly documented in nearly 50% of the cases. Among potential predictors of response, high PDL1 expression appeared to predominate in Pit-1 derived neoplasia, and elevated tumor mutation burden and/or mismatch repair deficiency in T-pit derived tumors, although all were dispensable. Based on encouraging results and generally acceptable safety profiles, the potential role of ICIs in refractory PitNETs and ICIs regimens are discussed.

**Abstract:**

**Background**: Aggressive and metastatic PitNETs are challenging conditions. Immune checkpoint inhibitors (ICIs) are currently considered in cases resistant to temozolomide (TMZ). However, clinical experience is essentially limited to case reports, with variable outcomes. **Material and** **Methods**: The effects of ICIs on 12 aggressive/metastatic PitNETs from the literature were reviewed and analyzed according to tumor characteristics, with the additional description of a silent-Pit1 metastatic tumor responding to pembrolizumab. **Results**: Most cases were metastatic (10/13: 6 corticotroph, 3 lactotroph, 1 silent Pit1); 3 were aggressive (2 corticotroph, 1 lactotroph). ICIS was used either as monotherapy or in combination. At last follow-up on ICI, a complete response (CR) was present in 3 cases and a partial response (PR) in 2 cases (4/5 metastatic). One sustained stable disease (SD) was reported. Progressive disease (PD) was observed in 7 cases, 3 of them after initial SD (*n* = 1) or PR (*n* = 3), with 2 reported deaths. PDL1 expression was studied in 10 cases and was high (>95%) in 2 Pit1-derived metastatic PitNETs (1 CR and 1 remarkable PR) but absent/low (<1%) in the remaining cases (including 1 CP and 2 PR). Elevated tumor mutation burden could be informative in corticotroph PitNETs, especially in mismatch repair-deficient tumors. **Conclusion**: Significant benefits from ICIs were documented in about half of TMZ-resistant PitNETS. High PDL1 expression was associated with remarkable responses but may be dispensable. Based on their acceptable tolerance and awaiting recognized predictors of response, ICIs may be considered a valuable option for such patients.

## 1. Introduction

Metastatic pituitary neuroendocrine tumors (PitNETs), also called pituitary carcinomas, are rare and their treatment is currently similar to that of aggressive PitNETs, which are characterized by uncontrolled growth despite standard treatments including surgery, anti-secretory drugs, and radiotherapy [1]. Both represent clinical challenges and oral chemotherapy with temozolomide (TMZ) is currently accepted as the first option, as it may dramatically improve the prognosis of some tumors [1,2]. Overall, TMZ induces a partial or complete radiological response in about one-third of the cases and a significant increase in 5 years survival and progression rate, although re-challenge with TMZ in the presence of tumor regrowth after drug withdrawal is often disappointing [1]. If primary or secondary resistance to TMZ occurs, alternative therapeutic strategies are needed. These may include peptide receptor radionuclide therapy (PPRT), angiogenesis-targeted therapy, and immunotherapy [1].

Immunotherapy based on the use of immune checkpoint inhibitors (ICIs) has recently revolutionized the treatment of several solid tumors, demonstrating efficacy and favorable safety profile in different cancer settings [3] and representing a new milestone in oncology. ICIs are monoclonal antibodies targeting the programmed cell death protein 1 (PD1), the programmed death ligand 1 (PDL1), and/or the cytotoxic T lymphocyte-associated protein 4 (CTLA-4), enhancing the immune system response against tumor cells [4,5,6]. However, the objective response is variable and, among tumor characteristics, PDL1 expression, tumor microenvironment (TME)—including tumor-infiltrating lymphocytes (TILs), tumor mutational burden (TMB), microsatellite instability (MSI), and/or abnormalities of the mismatch repair (MMR) system have been proposed as potential predictive markers for personalized therapy [7,8].

Based on their potential clinical efficacy and acceptable tolerance, ICIs are currently under active evaluation in patients with neuroendocrine tumors (NETs) of different origin, based on either single or combined therapeutic protocols [9,10,11,12,13,14]. In PitNETs, the experience is yet limited to case reports or small case series reported since 2018 [15,16,17,18,19,20,21,22,23], including aggressive and metastatic tumors—which will be respectively designed as ag-PitNETs and met-PitNETs. All patients had previously received TMZ and the outcome of ICIs was variable despite very encouraging reports.

The aim of this study was to review in detail the individual characteristics of PitNETs treated by ICIs in light of the current literature, starting from our recent experience with a non-functioning met-PitNET derived from the Pit1 lineage. In this latter case, a very high expression of PDL1 prompted us to start a treatment with pembrolizumab (Pembro), and a remarkable response was observed. We therefore aimed to analyze the reported outcome of ICIs according to tumor features, with a special focus on their molecular characteristics. A second endpoint was the evaluation of ICIs tolerance according to the therapeutic schedule.

## 2. Clinical Observation: Efficacy of Pembrolizumab in A Metastatic Non-Functioning PitNET

**Figure 1:**
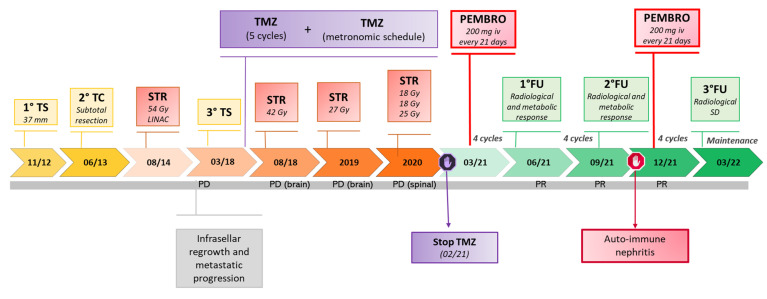
Timeline of disease progression and treatment in a silent Pit1 aggressive PitNET with a secondary metastatic dissemination: Metastatic dissemination was observed together with local progression after 2 neurosurgical operations—transsphenoidal (TS) and transcranial (TC), and an initial response to post-operative stereotactic radiotherapy (STR). After re-operation on the primary site (TS), temozolomide (TMZ) was first given as a classical fractionated schedule—340 mg/d, 5 days every 28 days—for 5 cycles, followed by a metronomic schedule (120 mg then 80 mg/d) for 30 months. Complementary STR was also given to the primary site and to multiple metastatic sites (brain and spinal). Pembrolizumab (Pembro) was started 200 mg/21 days in March 2021 and, after a short transient withdrawal because of auto-immune side effects including nephritis after cycle 8, is still ongoing at the time of the current report, with mild persisting cutaneous effects managed by discontinuous low-dose steroid therapy.

**Figure 2:**
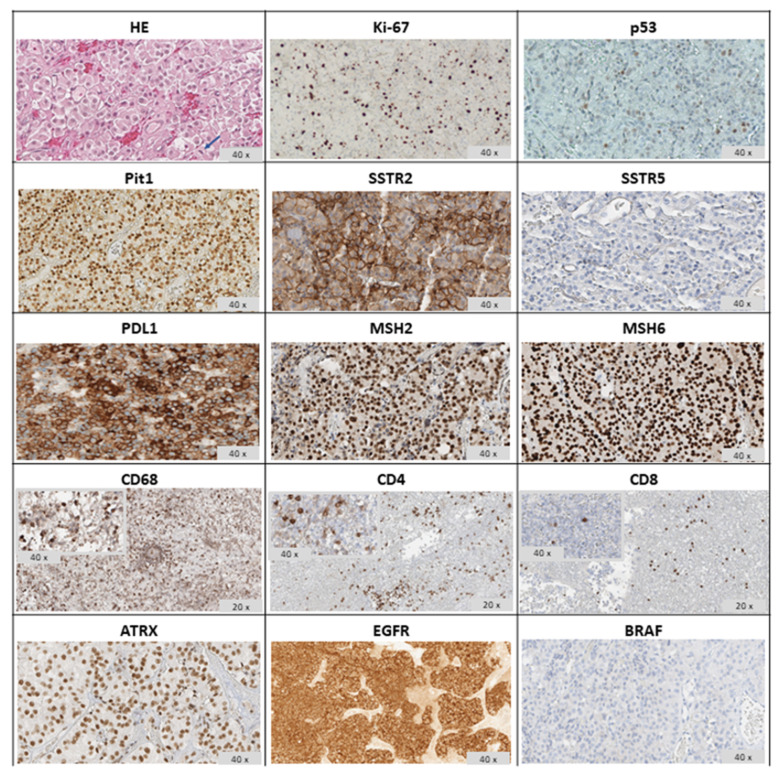
Pathological characteristics of the tumor at last surgery: Shown are mitosis on hematoxylin-eosin (**HE**) staining (indicated by an arrow); immunostaining for **Ki67** (20%) with positive **p53** staining (10%); diffuse nuclear **Pit1** immunoreactivity; positive **SSTR2** immunoreactivity and negative **SSTR5** expression; diffuse **PDL1** expression in neoplastic cells (95%), diffuse nuclear **MSH2** and **MSH6** expression; tumor-infiltrating inflammatory cells consisting of a predominance of macrophages (**CD68**+), with **CD4**+ lymphocytes exceeding in number **CD8**+ lymphocytes. Other molecular markers include **ATRX**, revealing a diffuse nuclear immunoreactivity; diffuse **EGFR** staining with some membranous enhancement, and negative **BRAF V600E** staining. In addition (data not shown), immunostaining for pituitary hormones was negative as already observed at first surgery, the tumor presented an unmethylated pattern of the MGMT promoter and, as previously reported, no telomerase abnormalities [24].

**Figure 3:**
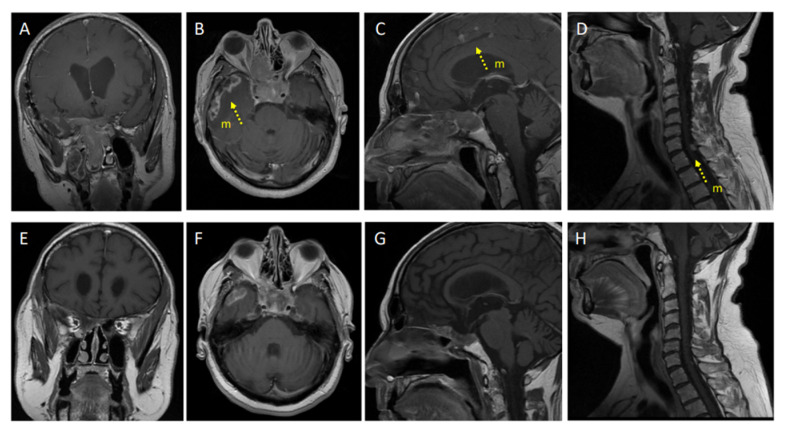
Radiological response to pembrolizumab: Shown are representative MRI obtained before (**A**–**D**) and after 1 year of treatment (**E**–**H**) in the patient affected by a silent metastatic Pit1-positive PitNET. Coronal (**A**,**E**) and axial (**B**,**F**) T1-weighted sequences post-gadolinium, show a regrowth of the sellar mass inferiorly with nasal obstruction (**A**), with anterior spread to the right retrobulbar optic area (**B**), and tumor shrinkage (>70%) after pembrolizumab with the persistence of minimal tissue of uncertain significance at the primary site (**E**,**F**). Sagittal T1-weighted sequence post-gadolinium (**C**,**G**) showing brain dissemination with the appearance of multiple, small and asymptomatic multiple metastases (m, yellow arrow)—the necrotic aspect of a metastatic site which previously received stereotactic radiotherapy along with metronomic temozolomide can also be recognized in (**C**), and their subsequent disappearance after pembrolizumab. (**G**) Spinal sagittal T1-weighted sequence post-gadolinium (**D**,**H**) showing the progression with the main spinal metastasis at C6–D1 (**D**) (yellow arrow) which nearly disappeared after treatment (**H**).

**Figure 4:**
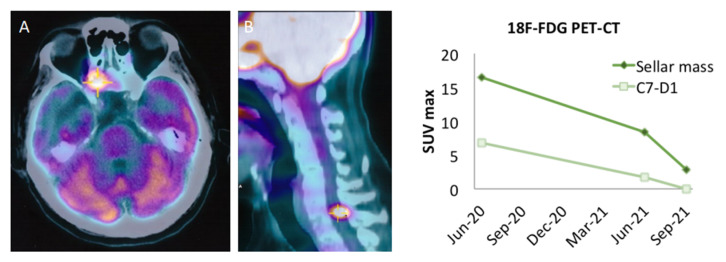
Metabolic response to pembrolizumab**:** Shown is representative imaging obtained by ^18^FDG-PET-CT before treatment ((**A**), sellar/parasellar mass; (**B**) main spinal metastasis C7–D1) and the metabolic response, illustrated by the evolution of SUV max at ^18^FDG PET-CT before and after 4 and 8 cycles of treatment.

The clinical history of our patient is summarized in Figure 1. He first came to our observation at the age of 57 yr for a large non-functioning intra/suprasellar mass (maximal diameter 37 mm), revealed by bitemporal hemianopsia, and invasive into the left cavernous sinus; basal pituitary function was normal, including normal prolactinemia. He underwent transsphenoidal (TS) surgery in November 2012 (Neuromed, IRCCS, Pozzilli, Italy). A diagnosis of “null cell” PitNET was made based on negative immunostaining for all pituitary hormones, with no mitosis but a focally high Ki67 (10%) and a low p53 expression (5%). In June 2013, the surgical resection was completed transcranially, with similar pathological findings. Visual defects normalized and a small remnant was left in the left cavernous sinus (<10 mm). One year later, he received stereotactic radiotherapy (STR, 50.4 Gy) on the left cavernous due to initial regrowth (adjacent intrasellar and intra-cavernous nodules, 13 and 14 mm respectively), with a good response and shrinkage to a unique, small paracavernous lesion (11 mm) in 2015. However, infrasellar regrowth occurred in 2017, first presenting as nasal pseudopolyps, and leading to TS re-operation in March 2018. The pathological diagnosis was consistent with the aggressive clinical behavior (1 mitosis/10 HPF, Ki67 20%, p53 10%) and two small pre-pontine nodules revealed metastatic progression. Further immunohistochemical characterization of the tumor indicated a Pit1 lineage since first surgery. The patient started TMZ with a standard schedule for 5 cycles, together with complementary STR on the primary site and pre-pontine nodules, with an initial response. Then, additional, small, and asymptomatic brain metastases were detected at MRI, and TMZ was shifted to a metronomic schedule. In July 2020, a spinal lesion was also detected (C7-D1). In addition, the primary tumor was rapidly regrowing in the sella (maximal diameter 24 mm) and extended laterally and again inferiorly to the sphenoid sinus and nasal cavities, causing nasal obstruction, and anteriorly to the right retrobulbar optic area, causing homolateral visual loss. For such reasons, complementary STR was given to the frontal and temporal areas, emerging brain metastasis, and the C7-D1 spinal lesion, during metronomic TMZ treatment (2019–2020). Metabolic re-evaluation by 18FDG PET-CT confirmed disease progression with a sellar/suprasellar, nasal, and spinal uptake, whereas the small, irradiated brain metastases were negative and showed necrotic changes at MRI. Searching for alternative therapeutic options, a high expression of PDL1 was found by immunohistochemistry (95%) suggesting immunotherapy targeting the PD1/PDL1 pathway. The pathological and molecular characterization of the tumor at last surgery is shown in Figure 2 (see legend for details). In the meantime, further asymptomatic spinal dissemination was noticed (December 2020) and nasal obstruction worsened, causing progressive respiratory distress. TMZ was therefore withdrawn and in March 2021 the patient started Pembro as an off-label treatment, according to a standard schedule (200 mg i.v every 21 days), upon approval by the Ethic Committee of Sapienza University of Rome (NETs unit). The patient reported an improvement in nasal obstruction, breathing, and vision since the 2nd cycle of treatment. Nasal and respiratory symptoms had resolved, and visual autonomy had recovered, when, after 4 cycles of treatment, significant tumor shrinkage was confirmed by MRI (>20% of the sellar/parasellar mass, 50% of the pre-pontine and spinal metastases), accompanied by a reduction in SUV max (8.5 vs. 16.5) of the primary tumor and main spinal metastasis (1.7 vs. 6.8) at 18FDG PET-CT. Further evaluation was obtained after four additional cycles of Pembro, showing a remarkable radiological and metabolic response, with: (i) a further shrinkage of the primary tumor (>70%); (ii) the disappearance of most brain and spinal metastases, with a necrotic aspect of small residual lesions, and the absence of new metastatic sites; (iii) a further decrease in SUV values of the primary tumor and the major spinal lesion (to 2.9 and 0, respectively). The radiological and metabolic response to treatment is illustrated in Figure 3 and Figure 4. Moderate cutaneous and renal toxicities (G1–G2) occurred, including a maculo-papular skin rash since the first cycles, a transient mild hypereosinophilia, and an acute increase in creatinine up to 2.4 mg/dL with fatigue and increased blood pressure at cycle 8, suggesting auto-immune nephritis. These were successfully managed by low-dose systemic steroid therapy, except for the acute nephritis which required transient drug withdrawal at cycle 9 with a normalization of renal function. Pembro was therefore re-introduced and is still in use as a maintenance therapy, with a clinical and neuroradiological stabilization of the disease confirmed at last follow-up (March 2022, one year of treatment). Albeit remarkable and nearly complete, the response was classified as partial due to the persistence of minimal metabolic activity and imaging of uncertain significance at the primary site. From an endocrinological point of view, the patient developed progressive hypopituitarism since 2013 and was already on replacement therapy (cortisone acetate, L-thyroxine, and transdermal testosterone) when immunotherapy was started. The treatment was then maintained with transient increases in adrenal replacement therapy as required and a modest increase in L-thyroxine due to worsening central hypothyroidism in the absence of anti-thyroid antibodies.

## 3. Analysis of PitNETs Response to ICIs according to Their Functional and Aggressive/Metastatic Behavior

**Figure 5:**
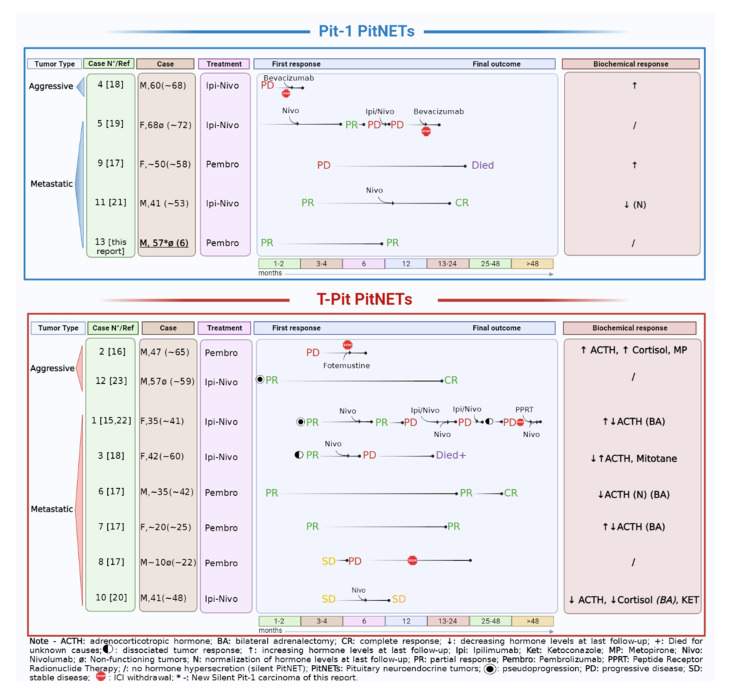
Graphical representation of the current experience with immune checkpoint inhibitors (ICIs) in refractory PitNETS including the new silent Pit1-positive metastatic PitNET reported herein. *Created by Biorender* [15,16,17,18,19,20,21,22,23].

Thirteen reported cases were reviewed, including 12 from the literature (using PubMed and Scopus up to mid May 2022) and the above observation. Overall, most were metastatic (10/13) and 3 were aggressive. Individual characteristics of the patients are reported in Table 1, with identifying case numbers which will be referred to in the text. All had previously received multiple treatments including repeated neurosurgery and radiotherapy for the primary tumor (and metastases in 6 cases), TMZ for a variable duration (3 to 43 cycles, median 12.5 cycles), TMZ in association with capecitabine (CAPTEM) in 4 cases (2 to 7 cycles, median 5.5 cycles), or other systemic regimens based on chemotherapy and/or biological drugs (5 cases). Classifying the tumors according to their lineage of origin, most were derived from the Tpit/corticotroph lineage (8/13, 61.5%), the others were from the Pit1 lineage (4 lactotroph and 1 silent Pit1 positive), and none were of gonadotroph origin. At the time of immunotherapy, 4 were clinically non-secreting (2 silent corticotroph, 1 silent lactotroph and 1 silent Pit1 positive), 3 were functional lactotroph PitNETS, whereas among the 6 patients with Cushing’s disease (CD), 4 had previous bilateral adrenalectomy (BA), including one consistent with Nelson’s syndrome (case 1), and one with persisting adrenal tissue and ongoing hypercortisolism (case 10). Some patients had also additional local treatments for metastatic lesions before immunotherapy was started, either as a combination of Ipilimumab (Ipi) and Nivolumab (Nivo) in 7 patients (dual ICI) or as a monotherapy with Pembro in 6 cases (mono ICI). Individual responses to treatment are illustrated in Figure 5. Because some responses changed with time, the final response status has been considered at last follow-up on ICIs.

All cases were considered refractory based on failure or escape to previous treatment with temozolomide, after multiple surgeries and irradiation to the primary and/or metastatic sites. The radiological and biochemical responses to ICIs are summarized according to tumor phenotype and behavior (aggressive vs. metastatic). Case numbers refer to Table 1 with reference to original reports; patient’s age is indicated at diagnosis and within brackets at the time of ICIs: dual regimen with ipilimumab and nivolumab (Ipi-Nivo) or monotherapy with pembrolizumab (Pembro).

A complete response (CR) was reported in 3 cases (1 metastatic lactotroph and 1 metastatic corticotroph, both with hormone suppression to undetectable levels, and 1 silent aggressive corticotroph) (cases 6, 11, and 12) [17,21,23]. A partial response (PR) was present in 2 metastatic PitNETs (1 corticotroph, with a decrease in ACTH secretion and 1 silent-Pit1) (cases 7 and 13) [17] and this report. In addition, one patient affected by CD with a met-PitNET experienced sustained tumor stabilization (SD) (12 months) with reduced ACTH and cortisol secretion (case 10) [20]. Taken together, these data indicate that 6/13 patients had significant clinical benefits from ICI without a need for additional treatment (46.1%).

In contrast, progressive disease (PD) was reported in 7 patients [15,16,17,18,19,22]. These included 3 cases of uncontrolled progression despite treatment at first evaluation (within 4 months) (1 aggressive corticotroph with CD, 1 aggressive prolactinoma, 1 metastatic prolactinoma) (cases 2, 4, 9). Out of them, one had early hyperprogression (case 4) and one died from PD (case 9), both had lactotroph tumors. In all cases, hormone secretion also worsened. Delayed PD was reported in 4 metastatic cases: in one silent corticotroph tumor, the patient came off the trial due to gradual symptomatic progression of the primary lesion after an initial stabilization (case 8), otherwise the escape occurred after PR at first evaluation in one silent lactotroph (case 5) and in 2 functioning corticotroph tumors (cases 1 and 3). PD was accompanied by increased hormone secretion in all functioning cases, and some authors suggested that uncontrolled hypercortisolism may reduce the efficacy of ICIs by reducing T cells activity [16,18]. Of note, delayed progression in corticotroph met-PitNETs was associated with dissociated responses among different tumor localizations: Duhamel et al. reported a significant metabolic and radiological response at the primary site and initial liver metastasis on dual ICI, with a new liver metastasis appearing during the same treatment, progressing upon Nivo as a maintenance therapy along with a slow regrowth of the primary mass (case 3). This patient died for unknown reasons despite a complete metabolic response of the new hepatic localization to radiofrequency ablation. Similarly, Lin et al. first reported PR at primary and liver metastases on dual ICI [15], then metastasis appeared at different sites (left adnexa and brain) [22] (case 1). At this point, additional doses of dual and mono- ICIs were given along with local treatments for metastases (surgery and/or STR) and PPRT (^177^Lu-DOTATATE) [22]. At last follow-up on Nivo monotherapy, the clinical picture was improving, with a substantial ACTH normalization and no new localization in the last 6 months [22].

We then analyzed the response to ICIs in patients starting on dual versus mono ICIs regimen. Among patients who achieved a CR, one started with mono ICI (case 6) and 2 with dual ICIs (cases 11 and 12). However, one of them was shifted to Nivo monotherapy and further improved from PR to CR (case 11). In other patients started on dual ICIs, the treatment was withdrawn for rapid PD and severe immune-related adverse effects (irAEs) in one case (case 4) or shifted to Nivo alone due to irAEs in 2 cases (cases 1 and 5). Of note, in these latter cases, a secondary escape occurred and re-challenge with dual ICIs alone failed, requiring additional local and systemic treatments [15,19,22]. In Case 1, pseudoprogression of liver metastasis was observed during 2 separate courses of dual ICIs [15,22]. On the other hand, among the 6 patients placed on Pembro alone, 2 had short-term PD (cases 2 and 9) and one had delayed PD after initial SD (case 8). The remaining 3 patients had long-term PR (cases 7 and 13) or CR (case 6). Overall, long-term clinical benefits from ICIs (without the need for additional treatments) were obtained in 3/7 cases starting with dual ICI and 3/6 cases starting with mono ICI. In addition, only 1/7 patients maintained long-term dual ICI (case 12).

## 4. Molecular Characterization of PitNETs and Potential Relationship with the Clinical Response to ICIs

Tumor molecular characteristics are reported in Table 1. PDL1 expression was clearly high (>95%) at the primary site in 2 met-PitNETs derived from a Pit1 lineage and showed a remarkable response to ICIs [21] and this report. In contrast, PDL1 was absent/low (<1%) in 8 cases, out of which 5 tested at the primary site [16,17,19] and 3 at a metastatic site (liver) [15,17,18]. Out of them, 3 were corticotroph met-PitNETs with CR (*n* = 1)/PR (*n* = 1) [17] or a dissociated response with subsequent PD [15,22]. In 3 cases, PDL1 expression was unavailable.

Mismatch repair deficiency (MMRd) was reported in 4 corticotroph PitNETs and associated either with CR (*n* = 2) [17,23], delayed PD (*n* = 1) [22] or rapid PD [16]. MMRd was diagnosed: (i) by IHC at the primary pituitary site in 2 aggressive cases [16,23], supported in one case by an MLH1 mutation [23]; (ii) at a liver metastatic site only, by IHC and supported by an MSH6 mutation [15]; (iii) at an orbital site harboring MSH2/6 mutations in a “hypermutated” phenotype [17]. In the remaining cases, no abnormality was found (*n* = 6) or searched for (*n* = 4). Alternatively, MSI was explored in 4 cases, but microsatellite stability (MSS) was found in all cases [17,18]. To review the data, tumor mutational burden (TMB) was re-defined according to Goodman (2017) as high (>20 mutations/megabase of DNA (mut/Mb), intermediate (6–19 mut/Mb), or low (up to 5 mut/Mb) [25]. Accordingly, TMB was high in a single case at a metastatic site [15] and intermediate in 3 cases—at primary site [23], metastatic site [17], or uncertain site [19]. In the first case [15], the high TMB observed at the liver metastatic site was associated with an MMRd state, which was in contrast with the absence of mutations at the primary site (before TMZ)—although both responded to dual ICIs—and with a low TMB at a further adnexal metastatic site appearing on maintenance ICIs—treated by surgery [22]. Among those with an intermediate TMB, one aggressive corticotroph PitNET was MMRd and had a CR [23], the others were either MMR-proficient or MSS and had PD. TMB was low in 2 additional PitNETs, an aggressive lactotroph [18] and a silent corticotroph met-PitNET [17], both were MSS and experienced PD. Therefore, the presence of a moderate or high TMB was associated with tumor response to ICIs in an MMRd setting, although this could be limited to metastatic sites.

## 5. Discussion

Our recent experience with a remarkable response to pembrolizumab of a met-PitNET expressing high levels of PDL1 prompted us to review and analyze the limited number of refractory PitNETs treated so far by ICIs and reported in the literature, in order to better understand the current benefits and limits of such treatment and the potential value of molecular markers as predictive factors for practical use in personalized medicine.

Including our observation, we could analyze 13 cases, all of them being clearly refractory to any current therapeutic option, including TMZ as recommended by recent guidelines [26]. This explains why all patients had severe clinical pictures, with a majority of metastatic tumors despite their rarity, and received ICIs as off-label options, with the exception of 5 cases issued from therapeutic trials [17,23]. Nonetheless, these cases were heterogeneous in terms of function, tumor burden, ICIs regimen, and molecular characteristics. Not surprisingly, most arose from the Tpit/corticotroph lineage, followed by the Pit1 lineage—the most common histotypes among ag-/met-PitNETs—and only 4 were clinically non-functioning at the time of ICIs therapy [17,19,23] and this report. Proliferative markers were not available in all cases but, at first pituitary surgery, 3/8 tumors (2 aggressive and 1 metastatic) had a low Ki67 index (≤3%) [16,18,20], whereas 5/8 were highly proliferative (Ki67 ≥ 10–80%) (2 aggressive and 3 metastatic) [18,19,21,23] and this report. Serial pathological characteristics also showed an increasing proliferation through subsequent surgeries in all available cases (3/3) [16,18] and this report. The Ki67 at metastatic sites was reported in 4 cases (10–50%) [15,18,19,21], and higher than at the pituitary site where available (3/4) [18,19,21]. Pathological and clinical similarities between ag- and met-PitNETs explain why they may be considered as “two sides of a same coin” and therefore benefit from similar therapeutic approaches, including ICIs [27].

The evaluation of tumor response to ICIs can be challenging and criteria may differ from those accepted for classical chemotherapy (e.g., RECIST = Response Evaluation Criteria in Solid Tumors) due to potential atypical responses such as “pseudoprogression”, “hyperprogression” or long-term stabilization of an aggressive disease [28]. Pseudoprogression is defined by an apparent increase in tumor size and/or development of new lesions, followed by a decrease in tumor burden; it may be observed in <10% of the patients [28]. This phenomenon may occur early (within the first 12 weeks of immunotherapy) or less frequently be delayed, and potentially leads to inappropriate ICIs withdrawal because of a misleading interpretation [28]. For such reasons, modified radiological RECIST criteria, such as the immune-related RECIST (irRECIST) or immune-modified (imRECIST), have been proposed [29,30,31] and early evaluation and follow-up (6–12 weeks or even 4–8 weeks) may allow recognizing such eventuality [28,29,31]. On the contrary, hyperprogression is defined by a rapid increase in tumor burden following the introduction of ICIs [28]. Although a minority of refractory PitNETS treated by ICIs were evaluated according to irRECIST [17,20], valuable information can be retrieved from the early experience in this field.

First of all, nearly 50% of treated patients had clear clinical benefits from ICIs, including CR (3/13), PR (2/13), and SD (1/13) at last follow-up. These results are encouraging compared with other attempts of molecularly targeted drugs in refractory PitNETs, similar to tyrosine kinase inhibitors (nearly 50%, CR 1/12, PR 5/12), better than everolimus (14.3%, SD 1/7), maybe worse than bevacizumab (CR 1/15, PR 4/15, SD 7/15) but in 4 cases (1 CR and 3 PR) the combination with TMZ complicate the interpretation of the data [1]. Moreover, with the exception of an early dramatic increase in ACTH secretion followed by a consistent decrease [17], a reduction in hormone secretion usually accompanied tumor response, sometimes achieving suppression to undetectable levels [17,21]. The effect on tumor volume and hormone secretion could translate into a significant improvement in general or neurological symptoms [15,18,19]. In our observation, visual and respiratory symptoms improved since the second cycle of Pembro. In such cases, very early radiological evaluation may be dispensable. However, the heterogeneity of tumor responses after the introduction of ICIs—rapid progression [18], pseudoprogression [15,23], dissociated responses [15,18,23], and delayed progression after PR [18,19,22] or SD [17]—strongly supports in most cases the need for an early radiological evaluation and appropriate follow-up, in order to avoid inappropriate premature discontinuation of ICIs.

The heterogeneity of response to ICIs reported in PitNETs points out the need for predictive factors of response, as in other solid tumors [32], although such attempts may be complicated by: (i) tumor heterogeneity, including the emergence of new metastases within the context of a primary or systemic response, leading to dissociated tumor response [19,22]; (ii) the absence of valuable pathological/molecular evaluation at the time of treatment. Indeed, as most refractory PitNETs have a longer clinical history as compared with other solid neoplasia, available tumor material may be outdated and no longer reflect the biology of the tumor. This is especially true if the last pituitary surgery was followed by radiotherapy or anti-neoplastic drugs, in particular TMZ, which may induce significant DNA modifications. Obtaining a “fresh” molecular characterization of primary or metastatic tissue—by surgical resection if indicated, or surgical biopsy if safe—may therefore be quite useful for clinical and research purposes. Potential predictive factors of tumor response to ICIs include PDL1 expression, MMR/MSI status, TMB, and TILs, of which clinical value is not universal and still represents a challenge in oncology [3,32]. Analyzing the current experience, an effort has been made in all cases to obtain molecular information, although not all the parameters cited above were studied simultaneously.

Immunohistochemical expression of PDL1 is the dominant tool used in clinical practice to inform about drugs targeting the PD1/PDL1 pathway. Indeed, PDL1 expression has been associated with clinical benefit across different tumor types [7], despite conflicting data that have been attributed to methodological issues (scoring system, antibodies, and platforms) and biological variability (transient expression, tumor heterogeneity) [7,19]. A higher expression of PDL1 has been reported in functioning (58.8%) than in non-functioning (34.3%) PitNETs [33,34] with a possible association with higher Ki67 LI suggesting therapeutic implications for functioning refractory PitNETs [34]. Larger studies have reported a lower proportion of PDL1 expression in PitNETs (15–18%), depending on the phenotype [35,36]. Early experience with the use ICIs in refractory PitNETs supports the concept that a high PDL1 expression (>95%) may be predictive of response, as it was associated with a CR to dual ICIs in a metastatic lactotroph [21] and a partial, nearly complete, response to mono ICI in a silent metastatic Pit-1 tumor (this report). These observations are also in agreement with data obtained on 264 PitNETs showing a preferential PDL1 expression in PitNETS derived from the Pit1 lineage (up to 60% vs. 0–6% in other phenotypes) [36]. Indeed, none of the corticotroph/Tpit-derived PitNET expressed PDL1 (<1% in all cases) [15,16,17,18]. However, because this did not preclude a clinical response to ICIs in some cases [17], patients should not be excluded from this option in the absence of tumor PDL1 expression.

TMB is the second most frequently explored predictive marker of response to ICIs in oncology [7]. Historically associated with resistance to chemotherapy, it may instead represent a positive predictive factor for immunotherapy due to the expression of multiple neoantigens eliciting an immune response [32]. TMB may be enhanced by tumor MMRd/MSI [32], and in June 2020, the Food and Drug Administration (FDA) approved the use of Pembro for patients affected by unresectable MMRd/MSI neoplasia. TMB may also be enhanced by the previous use of alkylating drugs, such as TMZ [37], which is of special interest for refractory PitNETs, which had all received TMZ as to current guidelines [1,15,16]. Similarly, radiotherapy may induce DNA alterations, especially if used with a large dose per fraction, and better outcomes have been reported when ICIs are combined with radiotherapy [38]. According to current experience with PitNETs, an elevated (high or intermediate) TMB was reported in 4 cases (2 on metastasis) and associated with variable responses including a CR [23]. According to early experience in PitNETs, an elevated TMB appeared to be predictive mostly in an MMRd context, but the impact of previous treatments is rarely valuable.

TILs are another potential predictive factor of response to ICIs, playing a critical role in the composition of the TME, which is in turn related to tumor characteristics and behavior [39,40]. TILs were found to strongly correlate with PDL1 expression in PitNETs [33,34]. In refractory PitNETs treated by ICIs, TILs were studied only in 2 cases, both reporting a high score (>2) in the absence of PDL1 (negative IHC), with variable outcomes (1 PR and 1 PD) [17]. In our case, tumor-associated macrophages predominated over lymphocytes, but little is known about their impact on PitNETs behavior and therapeutic response. Aggressive tumors may show a macrophage polarization toward an immunosuppressive and pro-tumoral subtype [41], but our patient had a remarkable response to immunotherapy. Of note, previous treatments may also modify the TME. If TMZ may deplete TILs and reduce PDL1 expression [19], radiotherapy may induce inflammation, attract TILs, and stimulate an immune response through the expression of PDL1, as described in sarcomas [42]. A combination of re-irradiation and TMZ treatment may be safe and efficacious in the local control of recurrent primary or secondary sites in recurrent ag-/met-PitNETs [43]. Where it fails, the combined effects of chemo-radiotherapy may therefore subsequently favor the response to ICIs through local inflammation, in addition to the DNA alterations mentioned before.

Immune-related adverse events (irAEs) often occur during ICI immunotherapy and vary according to the tumor and drug type and/or association [3], monotherapy being tolerated better than combined therapy [44]. They are typically more frequent in responsive patients, but their severity may represent a clinical limitation to ICIs. Depending on the severity, their treatment relies on corticosteroid therapy—which may decrease the efficacy of ICIs, especially in an adjuvant setting [45], and/or transient/definitive ICIs withdrawal. Thus, identifying predictive factors for severe irAE is another current oncological challenge [3]. Overall, the safety profile of ICIs in refractory PitNETs showed an acceptable tolerance, although dual regimen was shifted to monotherapy in several patients because of severe irAEs [15,19,20]. Among endocrine irAEs, the risk of immune-related central acute adrenal failure in patients receiving ICI for refractory PitNETs should be considered, although patients are generally already on adrenal replacement therapy and educated towards such events. Potential worsening of pituitary function due to hypophysitis has been rarely addressed in refractory PitNETs, but Lin et al. reported worsening of central hypothyroidism on dual ICI, as observed in our patient [15]. However, regular endocrinological evaluation is necessary to adapt the replacement therapy as appropriate due to the potential combined impact of repeated surgery, radiotherapy, and ICIs. Of note, shifting from dual to mono ICI could be followed by secondary escape in responsive cases [19,22], and re-challenge with dual ICI may not be sufficient to control the disease [22]. The first-line use of dual ICIs may be supported by the frequent expression of CTLA4 in PitNETs, regardless of tumor type [44,46], but this point was not addressed in refractory PitNETs. Overall, the current experience suggests that the favorable efficacy/safety profile of Pembro may place this drug as a first choice in refractory PitNETs, especially in the presence of some tumor characteristics—PDL1 expression or MMRd/elevated TMB.

To date, two registered phase II clinical trial studies are ongoing to better define the role of ICIs in ag-/met-PitNETs. NCT04042753 aims to determine the efficacy and safety of dual Ipi + Nivo in PitNETs after failure of surgery and radiation therapy. The study started in July 2019 with an estimated enrollment of 21 patients and an estimated completion date in July 2022; the current status is recruiting, and no preliminary data are available. NCT02834013 is also based on a dual Ipi + Nivo combination in people with rare tumors, including metastatic PitNETs (closed to accrual). The study started in July 2016 with an estimated enrollment of 818 patients and a completion date in October 2023. Data from non-pancreatic NETs cohorts of the trial have been recently published with encouraging results for high-grade neoplasms [47], but no data were available on met-PitNETs.

## 6. Conclusions

Few satisfactory treatments are currently available for refractory PitNETs, which are burdened by a high morbidity and mortality. Early experience with ICIs is encouraging, suggesting that they represent a promising option with an acceptable tolerance, especially in monotherapy regimens. Based on the high variability of responses, clinicians should be aware of some specificities in the interpretation of tumor evolution on treatment, and further experience is needed to better identify reliable predictors of response, and possibly of severe irAEs, in order to integrate ICIs in an algorithm for personalized medicine. Previous chemo-radiotherapy regimens may theoretically increase tumor response by promoting hypermutated states and/or inflammation, which is of interest in refractory PitNETs. Concerning predictive molecular predictors of response, differences are emerging according to tumor lineage, as PDL1 has a preferential expression in Pit-1 derived neoplasia, whereas elevated TMB and/or MMRd have been so far reported in T-pit derived tumors. Overall, one of these factors could be found in most cases achieving a partial, complete, or dissociated response to ICIs [15,17,19,22,23] and this report, which is encouraging. However, as their positive and negative predictive values cannot be defined at this early stage, ICIs cannot be currently precluded on a molecular basis. The potential contribution of TME composition in the response to ICIs has been poorly studied. Further in-depth characterization of refractory PitNETs is advisable for clinical and research purposes.

## Figures and Tables

**Table 1:** Characteristics of aggressive and metastatic PitNETs treated by immune checkpoints inhibitors (ICIs).

Ref	Sex, Age (years), Case Number	Histotype, Functional Status	Metastases	Previous Treatments *	ICI Drugs (Schedule)	Proliferative Markers	Potential Predictive Markers/Genetics
Lin A.L., 2018 [15]	F, 35, Case 1	Corticotroph metastatic PitNET, CD/Nelson’s syndrome	Liver	NS (4 times); RT (2 times sellar); Pas, Ket, Cab/Ket, Mif, Met; BA; CAPTEM (4 + 2 cycles); Systemic chemotherapy (2 cycles) *.	Ipi (3 mg/kg) + Nivo (1 mg/kg) every 3 weeks (5 cycles). Maintenance with Nivo (6 months).	Metastasis: Ki-67 up to 50%	Primary: No variant of interest (°) MSH6 IHC pos. no MSH6 mutation Metastasis: MGMT IHC pos. PDL1 < 1% MMRd (MSH6 IHC neg. MSH6 mutation) TMB: 93 mut/Mb (high) Both: No USP8 mutation.
			Left adnexa	RT (sellar) Metastasis surgery	Ipi + Nivo (4 cycles). Maintenance with Nivo.	N/A	TMB: 3.5 m/Mb (low)
2021 [22]			Brain (1) Brain (2)	RT (metastasis) RT (metastasis) PPRT	Ipi + Nivo (4 cycles). Nivo (6 months).	N/A	N/A
Caccese M, 2019 [16]	M, 47 Case 2	Corticotroph PitNET, silent→CD	/	-NS (3 times); -RT; -Pas; -TMZ (2 years)	Pembro (200 mg) (4 cycles).	Ki-67 3%, p53 pos **(NS1/2)** Ki-67 > 3%, p53 pos **(NS 3)**	PDL-1 IHC 0%. MMRd (MSH2, MSH6 IHC neg)
Duhamel C, 2020 [18]	F, 42 Case 3	Corticotroph metastatic PitNET, CD	Liver (5), suspected bone	NS (3 times); RT (3 sellar); Pas, Cab, Ket, Met, Mitotane; TMZ (10 + 3 cycles); Hydroxyurea (3 months).	Ipi (1 mg/kg) + Nivo (3 mg/kg) every 3 weeks (5 cycles). Maintenance with Nivo (every 2 weeks for 12 cycles).	**Primary:** Ki-67 2%, M = 0, p53 neg **(NS 1)**; Ki-67 5%, M = 5/10 HPFs, p53 2% **(NS 3)**. **Liver metastasis:** Ki-67 10%, p53 7%.	**Liver metastasis:** PDL-1 IHC neg.
	M, 60 Case 4	Aggressive Lactotroph PitNET	/	NS (3 times); RT; Cab, Pas; TMZ (6 cycles).	Ipi (1 mg/kg) + Nivo (3 mg/kg) every 3 weeks (2 cycles)—(withdrawal for severe toxicity and PD).	Ki-67 10%, M = 5/10 HPFs **(NS 1**); Ki-67 10–11%, M = 1/10 HPFs (**NS 2**); Ki-67 25%, M > 20/10 HPFs (**NS 3**).	MGMT promoter partially methylated 9–12%; MMS. TMB: 1 mut/Mb (low) Single gene abnormalities reported (°°);
Lamb L.S, 2020 [19]	F, 72 Case 5	Lactotroph (Pit-1+, PRL+) metastatic PitNET, silent	Dural/spinal	NS (3 times); RT (2 times sellar + metastasis); Metastasis surgery; TMZ (3 cycles).	Ipi (3 mg/kg) + Nivo (1 mg/kg) every 3 weeks (2 cycles). Nivo (3 mg/kg, twice weekly—17 cycles). Ipi + Nivo (4 cycles) − (withdrawal for severe toxicity and PD).	**Primary:** Ki-67 10%, M = 5/10 HPFs (**NS1**) **Metastasis:** Ki-67 20%.	**Primary (?):** PDL-1 IHC < 1%; “MMR proficient”; TMB: 6.8 mut/Mb (intermediate). **Metastasis:** MGMT IHC pos.
Majd N, 2020 [17]	M, mid-30 s Case 6	Corticotroph metastatic PitNET, CD	Liver, retroperitoneal lymphnodes, SNC	NS (3 times); RT (3 times sellar + metastases); BA; TMZ (16 + 8 cycles); CAPTEM (1 + 4 cycles); Local treatment of metastasis including surgery; FGFR inhibitor (2 cycles); CCNU + Bvz (1 cycle)	Pembro (200 mg) for 29 cycles.	N/A	**Orbital localization** MMRd.: “Hypermutated” phenotype, including MSH2, MSH6 and FGFR4 mutations. **Liver metastasis (IHC):** PDL-1 neg; TIL score 2
	F, early 20 Case 7	Corticotroph metastatic PitNET, CD	SNC, Bone, liver, pleura	NS (2 times); RT (sellar); BA; Pas; TMZ (7 cycles); CAPTEM (7 cycles)	Pembro (200 mg) for 15 cycles.	N/A	**Primary before TMZ:** PDL-1 IHC neg; MSS.
	M, late teens Case 8	Corticotroph metastatic PitNET, silent (ACTH+)	Dural, bone	NS (4 times); RT (2 times sellar + metastases); TMZ (12 + 7 + 2 cycles); IDO1 pathway inhibitor (11 cycles).	Pembro (200 mg) for 6 cycles.	N/A	**Primary:** PDL-1 IHC neg. MSS. TMB: “low” TIL negative.
	F, early 50 Case 9	Lactotroph metastatic PitNET	Bone, liver	NS; RT (sellar + metastases); Cab; Systemic chemotherapy (1 cycle) **; TMZ (12 + 2 cycles); CAPTEM (2 cycles).	Pembro (200 mg) for 6 cycles.	N/A	**Tumor (Primary?):** PDL-1 IHC neg; TIL score 2. **Metastases:** No mutation (bone) MSS (liver); TMB: “intermediate” (liver).
Sol B, 2021 [20]	41, M Case 10	Corticotroph metastatic PitNET, CD	SNC and spine	NS (2 times); RT (2 times sellar); Ket, Pas, Cab; BA; TMZ (3 + 9 cycles).	Ipi (3 mg/kg) + Nivo (1 mg/kg) every 3 weeks (4 cycles). Maintenance with Nivo (240 mg) every 2 weeks.	Ki-67 < 1%, p53 pos (1+) (**NS 1**)	N/A
Goichot B, 2021 [21]	41, M Case 11	PRL-secreting metastatic PitNET	Lung, pancreas, SNC	NS (2 times); RT (3 sellar + metastases); Cab; TMZ (43 cycles); Metastases surgery (2).	Ipi (3 mg/kg) + Nivo (1 mg/kg) every 3 weeks (4 cycles). Maintenance with Nivo every 2 weeks.	**Primary:** Ki-67 40% (**NS 1**). **Lung metastasis:** Ki-67 40–50%.	PDL-1 IHC 95% (sphenoid).
Shah, 2022 [23]	57, M Case 12	Sparsely granulated corticotroph aggressive PitNET–No CD (ACTH+)	/	NS; RT (sellar); TMZ (3 cycles).	Ipi (3 mg/kg)+ Nivo (1 mg/kg) every 3 weeks for 4 cycles. Maintenance Nivo (480 mg every 4 weeks) for 10 cycles.	Focal Ki-67 75–80%, moderate p53 pos. (NS1)	MGMT promoter methylation; MMRd: MLH1 and PMS2 IHC neg; MSH2 and MSH6 IHC pos; MLH1 and TP53 mutations; TMB: 8.8 mut/Mb (intermediate)
Feola, 2022 [this report]	57, M Case 13	Pit-1 non-functioning metastatic PitNET (Hormone negative)	SNC and dural	NS (3 times); RT (2 times sellar + metastases); TMZ (5 cycles+ metronomic schedule).	Pembro (200 mg) every 21 days.	Ki-67 10%, p53 < 5% (**NS 1/2**); Ki67 20%, p53 10%, M = 1/10 HPFs (**NS 3**).	**Primary:** MGMT promoter unmethylated; PDL1 IHC pos (95%); MSH2/6 IHC pos; CD68 > CD4 > CD8 IHC pos. TIL 1

Legend: *: Previous treatments are meant to summarize the complex management of the diseases before immunotherapy. BA: bilateral adrenalectomy; Bvz: bevacizumab; Cab: cabergoline; CAPTEM: capecitabine and temozolomide; CCNU: lomustine; CD: Cushing’s disease; HPFs: high-power fields; ICI: immune checkpoint inhibitor; IHC: immunohistochemistry; Ket: ketoconazole; Met: metyrapone; MGMT: O6methylguaninmethyltransferase; Mif: mifepristone; MLH1: MutL homolog 1; MMRd: mismatch repair deficient; MSH-2/6: MutS homolog 2/6; MSI: microsatellite instability; MSS: microsatellite stable; mut/Mb: number of mutations per megabase of tumor DNA; N/A: not available; NS: neurosurgery; Pas: pasireotide; PitNET: pituitary neuroendocrine tumor; PD: progressive disease; PDL-1: programmed death ligand 1; PPRT: peptide receptor radionuclide therapy; PMS2: postmeiotic segregation increased 2; RT: radiotherapy; Systemic chemotherapy: (*) Carboplatin/Etoposide, (**) Cisplatin/Etoposide TIL: tumor infiltrating lymphocytes; TMB: tumor mutational burden; TMZ: temozolomide; TIL: tumor-infiltrating lymphocytes score; (°) no somatic variant of interest (MSK-impact panel); (°°) *HGF* gene amplification; *CDKN2A/B* gene deletion; *BCORL1, FLCN*, and *SF3B1* genes mutations.

## Data Availability

Data concerning the illustrating case are available at Neuromed, IRCCS, Pozzilli (Italy).
